# First detection of bluetongue virus serotype 14 in Poland

**DOI:** 10.1007/s00705-016-2857-0

**Published:** 2016-04-11

**Authors:** Anna Orłowska, Paweł Trębas, Marcin Smreczak, Anna Marzec, Jan F. Żmudziński

**Affiliations:** Department of Virology, National Veterinary Research Institute, Partyzantów 57 Avenue, 24-100 Puławy, Poland

## Abstract

Here, we present the first detected cases of bluetongue virus (BTV) in native cattle from Poland. The virus was found in animals located near the Polish-Belarusian and Polish-Lithuanian borders. The positive animals were detected through an official epidemiological surveillance program. A combination of type-specific real-time RT-PCR and phylogenetic tests revealed the presence of BTV serotype 14 (BTV-14). This serotype is highly homologous to the vaccine strain and BTV-14 present in Russia, Lithuania, and Spain (from an animal imported from Lithuania). The most probable route of virus introduction to Poland was transmission through midges. All of the cases were subclinical.

Bluetongue virus (BTV) causes direct economic losses due to overt clinical disease in animals. The presence of BTV in ruminants restricts the international trade of animals [9]. BTV spread depends on circulation among ruminants, and it is transmitted by vectors that include several species of midges of the genus *Culicoides* [14]. BTV was previously considered an exotic disease in Europe, with only a few sporadic cases prior to 1998, e.g., a BTV-3 outbreak in Cyprus, BTV-4 in Turkey and Greece, and BTV-10 in the Iberian Peninsula [7, 11]. However, since 1998, different BTV serotypes including 1, 2, 4, 6, 8, 9, 11 and 16 have been continuously recorded in several Mediterranean regions, including European Union (EU) member states [16]. In August 2006, the first BTV cases were identified in northwestern Europe in domestic ruminants infected with BTV serotype 8 [4, 15]. Subsequently, BTV-6, BTV-11 and BTV-14 isolates that are genetically similar to vaccine strains have been found in northern Europe [1, 2, 8]. However, the route of their introduction is unclear. Poland remained free from BTV infections until 2012.

The Polish Veterinary Law of 2004 and the EU Regulation No. 1266 (passed in 2007) require active monitoring for BTV. From 2006 to 2008, the disease monitoring was for BTV clinical cases. This monitoring strategy did not detect any BTV infection outbreaks. The Chief Veterinary Officer (CVO) of Poland ordered testing by serology and RT-PCR for all animals imported from the member states with BTV problems. As a result, BTV-8 RNA was detected in 38 blood samples collected from German and Dutch animals imported to Poland between September 2006 and August 2008 [5]. All of the positive animals were slaughtered. In 2009, an active monitoring BTV program was introduced. Sera are obtained from cattle, sheep, and goats. The monitoring involves sample collections in May, July, and September. These months are associated with the highest midge activity. Between 2009 and 2010, three BTV-seropositive cattle and twelve seropositive sheep were identified using ELISA (ELISA kit Ingezim BTV DR 1.2.BTV.K.O from Ingenasa, Spain). The results refer to six administrative units (voivodeships) comprising 37.5 % of Poland and approximately 50 % of the cattle (approx. 3.2 million heads) and sheep (approx. 146 thousand heads) in the national herd. The seropositive animals were slaughtered. The follow-up survey of the herd was negative. In the subsequent year, 2011, a total of 1963 blood serum samples (1617 collected from cattle, 329 from deer, 13 from sheep and four from bison) from different regions of Poland were tested by ELISA (Ingezim, Ingenasa). Antibodies against BTV were detected in 494 animals (25.1 %), including 488 cattle (24.8 %), four deer (0.2 %) and two sheep (0.1 %). EDTA blood samples were taken from all of the seropositive animals and tested by real-time RT-PCR [13]. No positive results were found in a total of 494 samples.

A calf born on September 13, 2011 on a farm in Podlaskie voivodeship, Sokolski region, Miedzianowo village (Fig. 1) was sold in Spain on October 17, 2011. The calf was subsequently tested for BTV in Spain in December and had a positive BTV result. Before reaching its final destination, the calf was transferred three times between various holdings in Spain during the season when the ambient temperature was still highly suitable for midge activity. Finally, the calf was placed in a group of 127 calves also originating from Poland. However, this was the only calf among the 128 animals that tested positive (both ELISA and rtRT-PCR). Further molecular investigation including serotype-specific RT-PCR testing revealed negative results for BTV 1, 2, 4, 6, 8, 9, 11, 16, 24 and 26. Sequence analysis of the fragment of 194 nt from virus segment 5 revealed 100 % sequence identity to strain 600572 BTV-14, available in the GenBank database (accession no. FJ713346) [12].

**Fig. 1:**
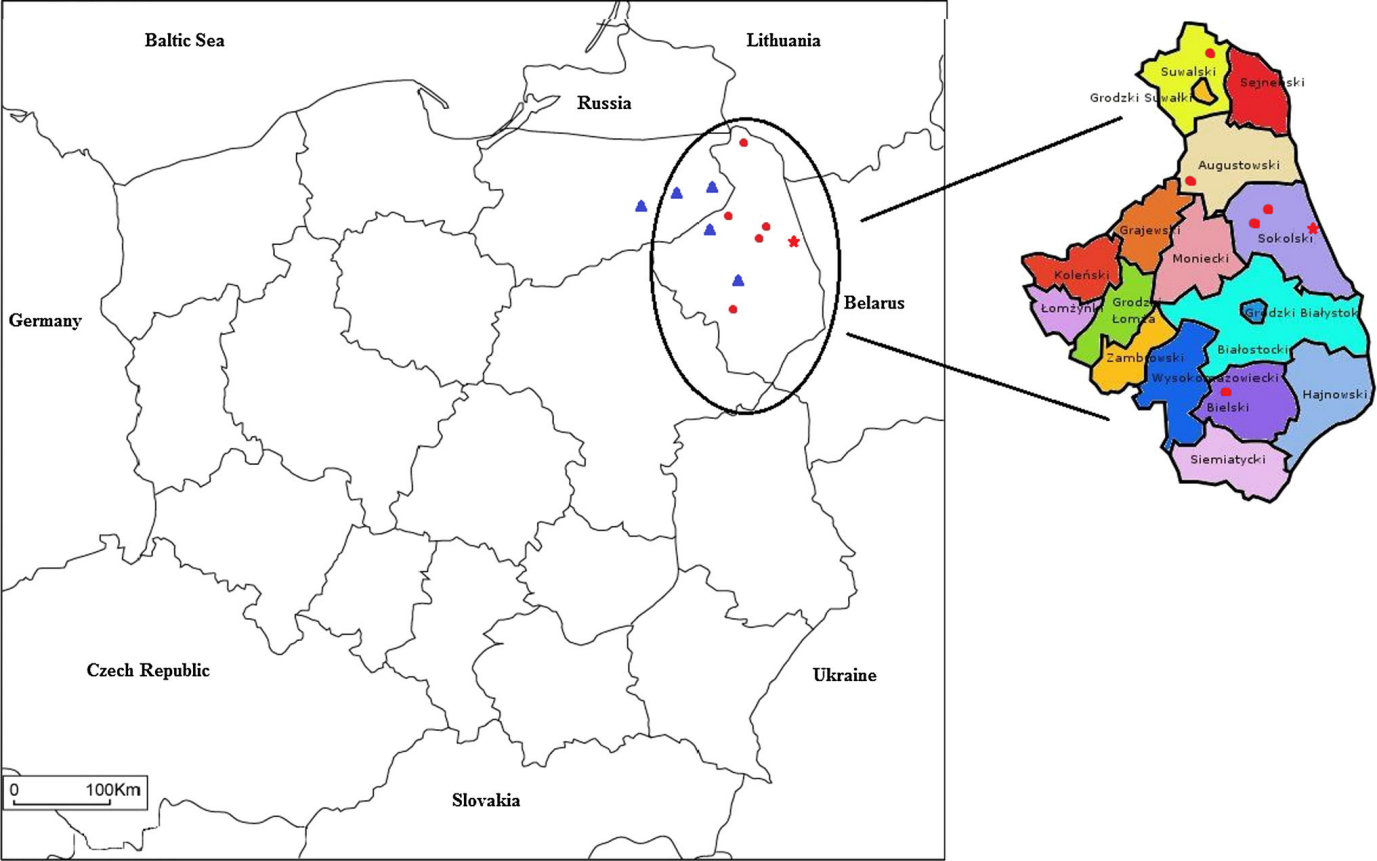
Map of Poland showing the distribution of BTV-14 serotype isolates collected in Poland. The indicated part of Poland shows the distribution of the BTV cases identified in 2012, indicated by circles. The star shows the location of Łowczyki village, the site of the first case of BTV in Poland. The triangles indicate subsequent BTV cases detected in 2014

In the same northeastern region of Poland (Podlaskie voievodeship, Sokolski region, Łowczyki village) in September 2012, BTV was diagnosed in cattle without clinical signs. Although the cattle lacked symptoms, the cases were identified due to the BTV surveillance program. In total, 19 animals were found seropositive, and four animals were rtRT-PCR BTV positive [13]. Furthermore, BTV genetic material was identified in cattle in the neighbouring administrative units (Suwalki region, Szypliszki village and Augustowski region, Bargłów Kościelny village) (Table 1). All of the animals were immediately slaughtered. The CVO requested further identification and virus typing, and blood samples were sent to the European Union Reference Laboratory for Bluetongue, Pirbright Institute, UK. According to the EU-RL protocol [8], the samples were tested twice by group-specific rtRT-PCR assays with the positive results of 24-33 Ct values. The serotyping results were negative for BTV 1, 2, 4, 6, 8, 11, and 16. The samples were also tested with a type-specific rtRT-PCR assay for BTV-14 (targeting segment 2, Seg-2), and the results were positive, with Ct values of 16-25. The rtRT-PCR typing results were confirmed by direct sequencing of the amplified products and phylogenetic analysis of BTV Seg-2, which represents the least conserved region of the BTV genome and thus the most suitable for molecular epidemiological studies [3]. It should be noted that the high homology between different isolates belonging to the common group/serotype of BTV-14 can be only ascertained for Seg-2. It is not possible to comment about sequences similarities in the other segments. Sequence analysis of Seg-2 (2922 bp) revealed that the BTV-14 serotype isolate belonged to the Western topotype of BTV-14. A comparison of the Polish BTV strain POL2012/01 showed 99.86 % nucleotide sequence identity to BTV-14 in the live attenuated vaccine strain from South Africa and 99.83 % nucleotide identity with the reference strain of BTV-14 [8]. The BTV-14 serotype has been circulating in western regions of Russia and the Smolensk region near the border of Belarus since 2011 [10]. The EU-RL report revealed a 99.79 % identity between the Polish BTV isolate and the BTV-14 isolate RUS2011/01 from Smolensk. Additionally, there was 99.83 % sequence identity between the Polish BTV isolate and the BTV-14 isolate SPA2012/01 collected in Spain from bovines imported from Lithuania. The isolates from Russia in 2011 and Spain in 2012 have 99.86 % and 99.76 % sequence identity to the vaccine BTV-14 strain. The high degree of sequence similarity between the Polish, Russian, and Spanish/Lithuanian strains and the vaccine strain may indicate the use of attenuated BTV-14 vaccine in the field. BT cases caused by vaccine strains (BTV-6 and BTV-11) were previously recorded in Europe. However, a live vaccine containing BTV-14 is not available on the EU market. Furthermore, vaccination against BTV is strictly forbidden by law in Poland. Additionally, the nucleotide sequences of the different strains collected from geographically distinct places are similar. Thus, the strains were derived from a common source and spread in the field. Phylogenetic comparison of whole genome sequences of BTV-14 isolates from different countries of Europe and isolated around the world revealed sequence similarities between BTV-14 European strains and existing vaccine and field BTV strains. Moreover, BTV-14 strains that have spread in Europe are reassortants containing genome segments derived from different reference vaccine strains originating from South Africa [6]. Because the virus is transmitted by midges, controlling the spread of BTV is difficult during periods of high *Culicoides* activity. We assume that BTV-14 appeared in Eastern Europe (possibly by illegal use of vaccine) and was spread to the West. The BTV-14 serotype appeared in 2011 in Smolensk at first. The following year, the virus was identified in Lithuania, Poland (but only the northeastern part of the country near Belarusian and Lithuanian borders, Fig. 1), and Spain, but only in an animal imported from Lithuania. BTV-14 infections in cattle have also been recorded in Latvia and Estonia. Animal movement as a route of virus transmission was excluded, as all of the animals imported into Poland were tested for the presence of BTV, and all rtRT-PCR-positive animals were slaughtered immediately. Thus, the introduction of BTV-14 by live animals was unlikely.

**Table 1:** Description of the origin of Polish BTV-14 isolates used in present study

Case	Collection date	Region	Village	Source	Age	Ct value
1	12.2011	Sokolski	Miedzianowo	Calf	3 months	
2	15.09.2012	Sokolski	Łowczyki	Cattle	-	31.66
3	15.09.2012	Sokolski	Łowczyki	Cattle	-	31.02
4	15.09.2012	Sokolski	Łowczyki	Cattle	-	36.95
5	15.09.2012	Sokolski	Łowczyki	Cattle	-	26.05
6	17.09.2012	Suwalski	Szypliszki	Cattle	6 years	25.96
7	17.09.2012	Augustowski	Bargłów Kościelny	Cattle	4 years	27.87
8	17.09.2012	Augustowski	Bargłów Kościelny	Cattle	10 years	26.53
9	27.01.2014	Białostocki	Zawyki	Cattle	3 years	29.18
10	27.01.2014	Białostocki	Zawyki	Cattle	6 years	30.08
11	25. 10.2014	Grajewo	Mierucie	Cattle	-	30.08
12	29. 10.2014	Olecko	Olecko	Cattle	5 years	29.69
13	29. 10.2014	Olecko	Olecko	Cattle	2 years	30.54
14	29. 10.2014	Mrągowo	Gązwa	Cattle	-	33.4
15	29.10.2014	Gizycko	Wyszowate	Cattle	-	32.62

Another possibility could be vehicles transporting live animals travelling through Poland (west-east and east-west). In the northeastern region of Poland, drivers stop to rest in a parking lot for lorries prior to crossing the Polish-Belarusian border. During this rest stop, midges could come into contact with BTV-positive animals transported from the east and transmit the virus to Polish animals located near the parking area.

Regarding the active monitoring programme for BTV, 11,405 blood serum samples were tested in Poland in 2012. One thousand nine hundred thirty-seven (17 %) out of a total of 11,405 samples were found to be seropositive in ELISA (Ingezim, Ingenasa), including 1915 cattle (98.9 %), 21 deer (1.08 %) and one sheep (0.02 %). Virological examinations [13] revealed seven BTV-14 cases located in four holdings in 2012 (see above).

At the beginning of 2014, two subsequent BTV-14 cases were diagnosed in bovines from one herd in Podlaskie voivodeship, Białostocki region, Zawyki village. Nine months later, in mid-October 2014, additional BTV-14 cases were identified in northeastern Poland. The virus spread to neighbouring Warmia-Mazuria voivodeship (Olecko, Mrągowo and Giżycko regions, Fig. 1). All of the infections were subclinical and were detected only under the obligatory monitoring program by routine commercial diagnostic tests for moving animals using the rtRT-PCR method [12]. Positive animals were slaughtered according to the decision of CVO.

From November 2014 to the end of December 2015, no BTV-14 cases were detected in Poland.
